# Analysis of Emergency Department Encounters Among High Users of Health Care and Social Service Systems Before and During the COVID-19 Pandemic

**DOI:** 10.1001/jamanetworkopen.2022.39076

**Published:** 2022-10-28

**Authors:** Melanie Molina, Jennifer Evans, Juan Carlos Montoy, Caroline Cawley, Dave Graham-Squire, Kenneth Perez, Maria Raven, Hemal K. Kanzaria

**Affiliations:** 1 Department of Emergency Medicine, University of California, San Francisco; 2 Benioff Homelessness and Housing Initiative, Center for Vulnerable Populations, University of California, San Francisco; 3 Provincial Health Services Authority, Vancouver, British Columbia, Canada; 4 Philip R. Lee Institute for Health Policy Studies, University of California, San Francisco

## Abstract

**Importance:**

Although the general US population had fewer emergency department (ED) visits during the COVID-19 pandemic, patterns of use among high users are unknown.

**Objectives:**

To examine natural trends in ED visits among high users of health and social services during an extended period and assess whether these trends differed during COVID-19.

**Design, Setting, and Participants:**

This retrospective cohort study combined data from 9 unique cohorts, 1 for each fiscal year (July 1 to June 30) from 2012 to 2021, and used mixed-effects, negative binomial regression to model ED visits over time and assess ED use among the top 5% of high users of multiple systems during COVID-19. Data were obtained from the Coordinated Care Management System, a San Francisco Department of Public Health platform that integrates medical and social information with service use.

**Exposures:**

Fiscal year 2020 was defined as the COVID-19 year.

**Main Outcomes and Measures:**

Measured variables were age, gender, language, race and ethnicity, homelessness, insurance status, jail health encounters, mental health and substance use diagnoses, and mortality. The main outcome was annual mean ED visit counts. Incidence rate ratios (IRRs) were used to describe changes in ED visit rates both over time and in COVID-19 vs non–COVID-19 years.

**Results:**

Of the 8967 participants, 3289 (36.7%) identified as White, 3005 (33.5%) as Black, and 1513 (16.9%) as Latinx; and 7932 (88.5%) preferred English. The mean (SD) age was 46.7 (14.2) years, 6071 (67.7%) identified as men, and 7042 (78.5%) had experienced homelessness. A statistically significant decrease was found in annual mean ED visits among high users for every year of follow-up until year 8, with the largest decrease occurring in the first year of follow-up (IRR, 0.41; 95% CI, 0.40-0.43). However, during the pandemic, ED visits decreased 25% beyond the mean reduction seen in prepandemic years (IRR, 0.75; 95% CI, 0.72-0.79).

**Conclusions and Relevance:**

In this study, multiple cohorts of the top 5% of high users of multiple health care systems in San Francisco had sustained annual decreases in ED visits from 2012 to 2021, with significantly greater decreases during COVID-19. Further research is needed to elucidate pandemic-specific factors associated with these findings and understand how this change in use was associated with health outcomes.

## Key Points

**Question:**

Did emergency department (ED) use decrease among the top 5% of high users of health care and social services in San Francisco County during the COVID-19 pandemic?

**Findings:**

In this cohort study of 8967 individuals, the rate of ED visits decreased by approximately 25% during the pandemic compared with nonpandemic years.

**Meaning:**

Factors associated with decreased ED encounters and health outcomes during the COVID-19 pandemic among previously high users are not clear and warrant further investigation.

## Teaser

This cohort study examines changes in the rate of emergency department visits among high users of health care and social services before vs during the COVID-19 pandemic.

## Introduction

High users of the health care system use a disproportionate amount of health services yet have poorer health outcomes.^1^ They are most commonly considered patients with 4 or more emergency department (ED) visits or 3 or more hospitalizations annually.^1,2,3,4^ Although high users as a group are heterogenous,^5,6,7^ they tend to have high rates of homelessness,^4,8^ substance use disorder,^9,10^ and chronic mental^1,10^ and medical illnesses^1,11,12,13,14^ and are often publicly insured.^5,12,13,14,15^ High users of health care services include frequent ED users, who often demonstrate high use of other services in addition to the ED, including ambulatory care services,^16^ mental health and sobering center visits, and psychiatric admissions.^11^ To better identify and prioritize individuals with fragmented care and high health care service use, the San Francisco Department of Public Health (SFDPH) developed a high users of multiple systems (HUMS) score, which incorporates use of urgent and emergency medical, mental health, and substance use services.^17^

To reduce health care costs and improve clinical outcomes, policy makers have become increasingly focused on targeting interventions toward high users.^11,18,19^ However, despite many attempts, few interventions have proven successful.^19,20,21^ Moreover, even without specific intervention, most high users do not sustain high use over time.^4,5,22,23,24,25,26,27^ For example, approximately 60% to 80% of frequent ED users in a given year do not exhibit frequent use in the next year,^5,22,23,25,26,27,28^ and ED use continues to decrease over time, leaving only a small group whose high use persists.^24^

The natural decay in service use observed in high users over time, commonly referred to as regression to the mean,^29^ makes it challenging to measure how use changes with specific interventions.^30^ Determining that a specific intervention is associated with reduced ED visits among high users without accounting for regression to the mean risks overestimating the association. Regression modeling techniques can control for the natural decay in use over time among high users, more accurately estimating the true association of interventions with health care use. A prior study^31^ used regression analyses to identify variables associated with high use and to estimate the risk of becoming a high user. However, to our knowledge, regression has not been used to model longitudinal service use among high users.

During the COVID-19 pandemic, fewer ED visits occurred across the nation, most prominently in the first few months.^32,33,34^ However, whether ED use among high users changed during the pandemic remains unclear. The pandemic resulted in decreased availability of some social services that support high users (eg, mental health, substance use, and housing services),^35,36,37^ whereas other services related to the pandemic (eg, isolation/quarantine hotels and managed alcohol programs)^38,39^ were increased. How ED use among high users changed during the pandemic is thus difficult to determine. If ED visits during the pandemic decreased among high users, identifying pandemic-specific factors that might be associated with this decrease could help inform future interventions; if the number of ED visits remained the same or increased, stronger efforts might need to be directed toward addressing high users’ complex needs in the ED setting during public health crises. Our goals in this study were (1) to describe the natural decay—commonly referred to as regression to the mean—in use among high users by using novel modeling techniques and (2) to leverage these innovative methods to determine whether ED use differed among high users during the COVID-19 pandemic.

## Methods

The Coordinated Care Management System (CCMS) is an SFDPH data platform that integrates patients’ medical and social information from multiple source systems. A CCMS record is created by the SFDPH for any person 18 years or older who meets at least 1 of the following conditions: listed as homeless in any San Francisco County health or housing system; uses county behavioral health, homelessness, or jail health services; and uses county urgent or emergency medical, mental health, or substance use services.^11,17^ The data are organized into yearly cohorts by fiscal year (FY), extending from July 1 of the starting year to June 30 of the following year. We obtained approval for research on partially deidentified human participants through the University of California, San Francisco’s institutional review board and adhered to the Protected Health Information and 42 *Code of Federal Regulations* (CFR) §2 protocols governing the use of substance use disorder data. In accordance with 45 CFR §46, informed consent was not obtained because the study did not involve direct contact with participants. This cohort study followed the Strengthening the Reporting of Observational Studies in Epidemiology (STROBE) reporting guideline.

As detailed elsewhere,^17^ the SFDPH uses CCMS data to calculate a HUMS score for each patient based on their use of 9 urgent or emergency services, which are categorized into medical, psychiatric or mental health, and substance use services (eTable 1 in the Supplement).^11,17^ To derive the HUMS score, all visits or stays to each service for each patient during the FY were counted. Each encounter contributed 1 point; services were not weighted and did not include length of stay. Patients whose total counts were within the top 5% were categorized as the top 5% of HUMS for that respective FY. We identified the top 5% of HUMS for each FY in our study (FY 2012 to FY 2020).

**Figure 1.**
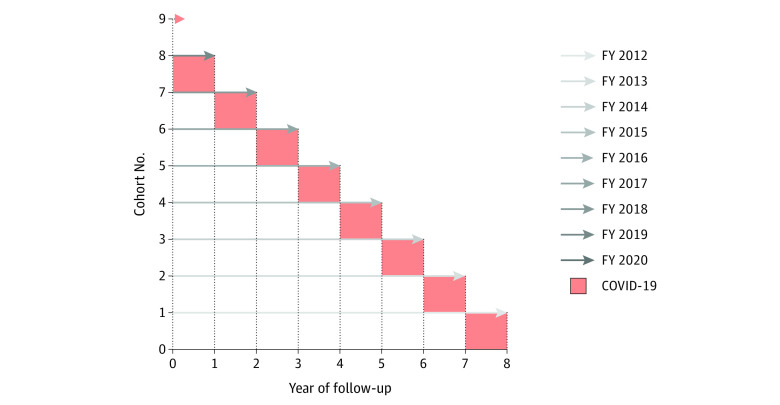
Nine Cohorts of the Top 5% of Higher Users of Medical Services (HUMS) Combined by Year of Follow-up Nine cohorts of top 5% of HUMS were defined. The prepandemic cohorts were followed up until fiscal year (FY) 2020, which was defined as the COVID-19 year (red). To account for the natural decay in ED visits among HUMS, the cohorts’ data were combined by year of follow-up rather than by temporal year. This allowed assessment of how ED visits during the COVID-19 year compared with the overall expected decay in ED visits among HUMS over time.

To assess ED visits among high users during the COVID-19 pandemic, we performed a retrospective cohort study of adults in the top 5% of all HUMS in San Francisco County during a 9-year period from FY 2012 to FY 2020. Each FY contributed its own cohort of unique top 5% of HUMS; thus, we had 9 cohorts for our analysis: 8 prepandemic cohorts and 1 pandemic cohort. We followed up the 8 prepandemic cohorts forward, from the first year they were defined as the top 5% of HUMS (year 0, the index year) to FY 2020 (the COVID-19 year) (Figure 1). Correspondingly, each cohort was followed up for a different amount of time, depending on its index year. For example, cohort 1 contained the top 5% of HUMS from FY 2012 (index year) and was followed forward for 8 years through FY 2020 (Figure 1). The final cohort 9 was defined in FY 2020 and therefore was not followed forward.

Because ED visits among high users decrease in the years immediately after the first year of high use,^24^ we combined each cohort’s mean annual ED visit counts by year of follow-up rather than temporal year. As a result, each cohort’s COVID-19 year (FY 2020) corresponded to a different follow-up year in the study period (Figure 1). Using the combined cohort data, we derived an overall prepandemic expected rate of ED visit decay during follow-up. We then compared the ED visit counts during each cohort’s COVID-19 year to the overall expected trend in ED visits among high users to evaluate whether ED visits during the pandemic decreased beyond the expected prepandemic decay. Given that the CCMS data were organized by FY and that most of FY 2019 (July 2019 through June 2020) was before COVID-19 in San Francisco County, we did not designate FY 2019 as a COVID-19 year.

For each cohort, we examined age, gender, language, race and ethnicity, history of homelessness, insurance status, jail health encounters (within the past year), Elixhauser categories of mental health and substance use diagnoses,^40^ and mortality. We collected and examined race and ethnicity as markers of differential experience of the health care system related to racism and to elucidate any existing inequities. Patients were classified as having a mental health or substance use disorder if there were 2 or more diagnostic codes that indicated the presence of these diagnoses in the index year or the 2 years prior.

### Statistical Analysis

We calculated numbers (percentages), means (SDs), and medians (ranges) for baseline measures of demographic characteristics, mortality, and missingness within each cohort. Given that the ED visit count data were overdispersed^41^ with a right-skewed distribution, we chose negative binomial over Poisson regression to model ED visits over time. We used a mixed-effects model because of the longitudinal nature of the study, which included repeated measures of each patient’s annual ED visit count.^42^ Fixed effects were the COVID-19 year indicator (FY 2020) and time, with time treated as a categorical variable corresponding to follow-up year (ie, index year = 0, year 1 = 1, and so on). The patient identifier variable was modeled as a random effect with patient-specific intercepts and slopes over time, using an unstructured covariance matrix. We checked for potential time-specific associations of the COVID-19 pandemic with annual ED visits by including a COVID-19 × time interaction term but found no evidence of a statistically significant interaction and thus excluded the term from the final model. We evaluated the association between ED visit counts with time and with COVID-19 by estimating incidence rate ratios (IRRs) with corresponding 95% CIs. Statistical significance was set at *P* < .05 (2-tailed).

Individuals were excluded from analyses if they died or if their last known contact with county services was more than 2 years in the past, suggesting they moved away or were lost to follow-up for other reasons. To examine data missingness, we compared demographic characteristics of participants who died or were lost to follow-up with those with complete follow-up using χ^2^ and Kruskal-Wallis tests for categorical and continuous variables, respectively. We performed sensitivity analyses using (1) a shorter follow-up period to minimize the number of patients who died or were lost to follow-up, (2) complete data only, and (3) data with ED visit counts replaced by 0 in participants lost to follow-up. All statistical analyses were performed using Stata MP, version 17.0 (StataCorp LLC).

## Results

Of 8967 unique study participants, 3289 (36.7%) identified as White, 3005 (33.5%) as Black, 1513 (16.9%) as Latinx, and 1046 (11.7%) as other race or ethnicity (114 [1.3%] declined to report race or ethnicity); 7932 (88.5%) preferred English as a primary language. The mean (SD) age was 46.7 (14.2) years; 6071 participants (67.7%) identified as men, 2814 (31.4%) identified as women, and 82 (0.9%) identified as transgender or other or declined to report gender. A total of 7042 participants (78.5%) had a history of homelessness (Table 1). Demographic characteristics were largely similar across cohorts, except for insurance status: participants from later cohorts had increasingly higher proportions of Medi-Cal. The mean (SD) ED visit count during the index year was 5.5 (5.7); the median was 5 (range, 0-138). A total of 1125 participants (12.6%) died, and another 1725 (19.2%) were lost to follow-up during the study period (eTable 2 in the Supplement).

**Table 1.** Demographic Variables by Cohort Index Year^a^

Variable	Total	Cohort index year								
		FY 2012	FY 2013	FY 2014	FY 2015	FY 2016	FY 2017	FY 2018	FY 2019	FY 2020
Age, mean (SD), y	46.7 (14.2)	47.7 (12.9)	47.1 (13.2)	47.3 (13.9)	45.7 (13.4)	46 (14.3)	46.1 (14.7)	46.4 (15.0)	47.3 (15.2)	46.5 (14.9)
Gender										
Men	6071 (67.7)	800 (66.2)	736 (68.4)	661 (67.9)	572 (68.3)	621 (68.2)	627 (67.8)	652 (66.3)	686 (69.8)	716 (66.9)
Women	2814 (31.4)	396 (32.8)	331 (30.8)	303 (31.1)	253 (30.2)	281 (30.8)	290 (31.4)	327 (33.3)	287 (29.2)	346 (32.3)
Transgender, other, or declined to respond	82 (0.9)	12 (1.0)	9 (0.8)	9 (0.9)	12 (1.4)	9 (1.0)	8 (0.9)	4 (0.4)	10 (1.0)	9 (0.8)
Language										
English	7932 (88.5)	1095 (90.6)	970 (90.1)	877 (90.1)	761 (90.9)	799 (87.7)	810 (87.6)	853 (86.8)	827 (84.1)	940 (87.8)
Spanish	699 (7.8)	73 (6.0)	73 (6.8)	55 (5.7)	50 (6.0)	74 (8.1)	84 (9.1)	89 (9.1)	104 (10.6)	97 (9.1)
Other	336 (3.7)	40 (3.3)	33 (3.1)	41 (4.2)	26 (3.1)	38 (4.2)	31 (3.4)	41 (4.2)	52 (5.3)	34 (3.2)
Race and ethnicity										
African American or Black	3005 (33.5)	416 (34.4)	349 (32.4)	348 (35.8)	293 (35.0)	322 (35.3)	304 (32.9)	326 (33.2)	289 (29.4)	358 (33.4)
Latinx	1513 (16.9)	173 (14.3)	179 (16.6)	141 (14.5)	122 (14.6)	144 (15.8)	165 (17.8)	183 (18.6)	194 (19.7)	212 (19.8)
White	3289 (36.7)	480 (39.7)	419 (38.9)	366 (37.6)	322 (38.5)	332 (36.4)	321 (34.7)	351 (35.7)	330 (33.6)	368 (34.4)
Other^b^	1046 (11.7)	116 (9.6)	114 (10.6)	112 (11.5)	83 (9.9)	93 (10.2)	124 (13.4)	114 (11.6)	160 (16.3)	130 (12.1)
Declined to respond	114 (1.3)	23 (1.9)	15 (1.4)	6 (0.6)	17 (2.0)	20 (2.2)	11 (1.2)	9 (0.9)	10 (1.0)	3 (0.3)
Homelessness^c^										
No	1925 (21.5)	244 (20.2)	196 (18.2)	214 (22.0)	147 (17.6)	212 (23.3)	207 (22.4)	243 (24.7)	239 (24.3)	223 (20.8)
Yes	7042 (78.5)	964 (79.8)	880 (81.8)	759 (78.0)	690 (82.4)	699 (76.7)	718 (77.6)	740 (75.3)	744 (75.7)	848 (79.2)
Last insurance status^d^										
Medicaid without SSI	3879 (43.3)	329 (27.2)	345 (32.1)	344 (35.4)	337 (40.3)	414 (45.4)	431 (46.6)	487 (49.5)	536 (54.5)	656 (61.3)
Medicaid with SSI/Medicaid/Medicare	3953 (44.1)	603 (49.9)	551 (51.2)	485 (49.8)	417 (49.8)	419 (46.0)	409 (44.2)	392 (39.9)	354 (36.0)	323 (30.2)
Medicare only	467 (5.2)	100 (8.3)	53 (4.9)	70 (7.2)	32 (3.8)	34 (3.7)	38 (4.1)	43 (4.4)	52 (5.3)	45 (4.2)
Other	668 (7.4)	176 (14.6)	127 (11.8)	74 (7.6)	51 (6.1)	44 (4.8)	47 (5.1)	61 (6.2)	41 (4.2)	47 (4.4)
Jail stay^e^										
No	6934 (77.3)	979 (81.0)	825 (76.7)	719 (73.9)	582 (69.5)	693 (76.1)	696 (75.2)	772 (78.5)	775 (78.8)	893 (83.4)
Yes	2033 (22.7)	229 (19.0)	251 (23.3)	254 (26.1)	255 (30.5)	218 (23.9)	229 (24.8)	211 (21.5)	208 (21.2)	178 (16.6)
Mental health diagnosis										
No	3450 (40.0)	471 (40.7)	440 (43.3)	364 (38.3)	270 (33.5)	356 (40.3)	326 (36.1)	375 (39.6)	388 (41.0)	460 (45.5)
Yes	5173 (60.0)	687 (59.3)	576 (56.7)	587 (61.7)	536 (66.5)	527 (59.7)	578 (63.9)	572 (60.4)	558 (59.0)	552 (54.5)
SUD diagnosis										
No	2791 (32.4)	412 (35.6)	352 (34.6)	293 (30.8)	213 (26.4)	278 (31.5)	318 (35.2)	327 (34.5)	283 (29.9)	315 (31.1)
Yes	5832 (67.6)	746 (64.4)	664 (65.4)	658 (69.2)	593 (73.6)	605 (68.5)	586 (64.8)	620 (65.5)	663 (70.1)	697 (68.9)
Follow-up										
Complete follow-up	6117 (68.2)	536 (44.4)	531 (49.3)	506 (52.0)	493 (58.9)	569 (62.5)	652 (70.5)	801 (81.5)	958 (97.5)	1071 (100.0)
Lost to follow-up^f^	1725 (19.2)	383 (31.7)	332 (30.9)	280 (28.8)	195 (23.3)	223 (24.5)	190 (20.5)	119 (12.1)	3 (0.3)	0
Died	1125 (12.5)	289 (23.9)	213 (19.8)	187 (19.2)	149 (17.8)	119 (13.1)	83 (9.0)	63 (6.4)	22 (2.2)	0

Abbreviations: FY, fiscal year; SSI, supplemental security income; SUD, substance use disorder.

^a^ Data are presented as number (percentage) of patients unless otherwise indicated.

^b^ Other includes Asian, Native Hawaiian or Other Pacific Islander, American Indian, Asian/Pacific Islander, Filipino, mixed or multiethnic, and an explicit “other” category.

^c^ History of homelessness.

^d^ Other includes uninsured, private insurance, Healthy San Francisco, or other insurers.

^e^ Within the past fiscal year.

^f^ Two years since last contact with San Francisco County services.

**Figure 2.**
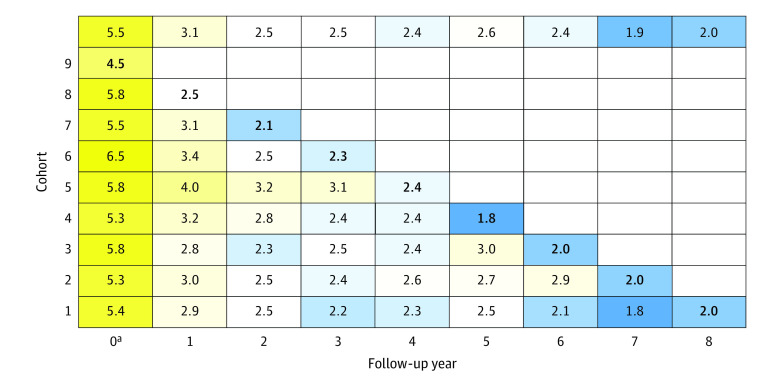
Heatmap of Mean Number of Annual Emergency Department (ED) Visits by Follow-up Year, Stratified by Cohort Yellow indicates greater mean number of ED visits; blue, fewer mean number of ED visits; boldface numbers, number of ED visits during the COVID-19 year. ^a^Index year.

Figure 2 shows the mean annual ED visits through time and across cohorts, aligning each cohort by year of follow-up. At the index year (year 0), all cohorts averaged more than 5 ED visits annually except cohort 9, whose index year was the COVID-19 year. The largest decrease in ED visits occurred between the index year and year 1, with continued but lesser decreases in subsequent years. When examining mean annual ED visits across cohorts by year of follow-up, the number of ED visits during the COVID-19 year tended to be less than during the non–COVID-19 years (eFigure 1 in the Supplement).

Table 2 gives the results of modeling the baseline, prepandemic decrease in the number of ED visits over time using data from all cohorts. The annual number of ED visits decreased most prominently in the first 2 follow-up years, with high users in year 1 after the index year having a 59% lower rate of ED visits (IRR, 0.41; 95% CI, 0.40-0.43) compared with those in the index year and high users in year 2 having a 34% lower rate of ED visits (IRR, 0.66; 95% CI, 0.63-0.69) compared with those in year 1. We also observed statistically significant decreases in annual ED visit rates in years 3 to 7 after the index year, but these decreases were less pronounced.

**Table 2.** Decrease in Number of Emergency Department Visits Over Time Before COVID-19 Year

Follow-up year	IRR (95% CI)	
	Compared with index year	Compared with previous year
1	0.41 (0.40-0.43)	0.41 (0.40-0.43)
2	0.27 (0.26-0.28)	0.66 (0.63-0.69)
3	0.22 (0.21-0.23)	0.80 (0.76-0.84)
4	0.18 (0.16-0.19)	0.81 (0.76-0.85)
5	0.15 (0.14-0.16)	0.85 (0.80-0.92)
6	0.12 (0.11-0.14)	0.82 (0.75-0.90)
7	0.09 (0.08-0.10)	0.71 (0.64-0.80)
8	0.09 (0.08-0.11)	1.07 (0.91-1.26)

Abbreviation: IRR, incidence rate ratio.

Compared with the non–COVID-19 years, the overall rate of ED visits during the COVID-19 year decreased by 25% (IRR, 0.75; 95% CI, 0.72-0.79) beyond the expected prepandemic year-to-year decrease. This finding was robust to sensitivity analyses using a shorter follow-up period, complete data only, and data with visit counts replaced with 0 in participants whose last known contact with county services was more than 2 years in the past. Table 3 gives the mean annual ED visits estimated by the model during each follow-up year, comparing non–COVID-19 and COVID-19 years. The model estimated fewer ED visits during the COVID-19 pandemic for each year of follow-up until year 7. For example, during year 1, the expected mean number of ED visits in the COVID-19 year (1.6; 95% CI, 1.5-1.7) was significantly less than that of the non–COVID-19 year (2.1; 95% CI, 2.0-2.2). The same was true of follow-up years 2 to 6 (eFigure 2 in the Supplement). These findings demonstrate that not only did the number of prepandemic ED visits decrease significantly from year to year at a decreasing rate, but during the pandemic, ED visit counts were also significantly less than expected at each year of follow-up through year 6.

**Table 3.** Mean Number of Emergency Department Visits Estimated by Mixed-Effects Negative Binomial Regression by Time and COVID-19 Year

Follow-up year	Mean No. of visits (95% CI)	
	Before COVID-19	During COVID-19
0^a^	5.1 (5.0-5.2)	3.8 (3.7-4.0)
1	2.1 (2.0-2.2)	1.6 (1.5-1.7)
2	1.4 (1.3-1.4)	1.0 (1.0-1.1)
3	1.1 (1.1-1.2)	0.8 (0.8-0.9)
4	0.9 (0.8-1.0)	0.7 (0.6-0.7)
5	0.8 (0.7-0.8)	0.6 (0.6-0.7)
6	0.6 (0.6-0.7)	0.5 (0.4-0.5)
7	0.4 (0.4-0.5)	0.3 (0.3-0.4)
8	0.5 (0.4-0.6)	0.4 (0.3-0.4)

^a^ Index year.

## Discussion

Measuring how health care use among high users changes with specific interventions has historically been challenged by a natural decay in service use over time, also referred to as regression to the mean.^30,43^ To circumvent this problem, we used mixed-effects, negative binomial regression to model ED visits over time among high users and then isolate the association of the COVID-19 pandemic with ED use beyond natural trends. In our study population, which had an overrepresentation of African American or Black and publicly insured individuals, statistically significant decreases occurred in ED visits both from year to year and during the pandemic.

Given that prior studies^44,45,46,47^ have used negative binomial regression to describe risk factors associated with high ED use, our use of the technique represents an advancement in modeling the natural trends in health care use among high users. The mixed-effects model allowed us to account for the longitudinal and clustered nature of the number of ED visits data. We could then make precise predictions regarding expected ED use and whether the COVID-19 pandemic was associated with a comparative change in use. Although we focused on evaluating ED visits during the pandemic, the same technique could be used to gauge an intervention’s association with use of other services while accounting for population-specific natural use trends.

Our study results are consistent with prior studies showing that approximately 60% to 80% of frequent ED users in a given year do not exhibit frequent use ED services in the next year.^5,22,23,25,26,27,28^ We extended this finding in 2 ways. First, by following up cohorts of high users for an extended period, and second, by studying multiple overlapping cohorts, we were able to model natural trends in health care use in this population, revealing a persistent yet decreasing rate of decay in ED use over time. Although several studies have examined ED use in the general population during the COVID-19 pandemic,^32,35,48^ we leveraged mixed-effects, negative binomial regression to describe health care use patterns during the pandemic specifically among high users. One Canadian study that examined hospitalizations in unhoused individuals during the pandemic found that, unlike the general population, these individuals did not experience a significant reduction in hospitalizations.^49^ Although the majority of our study population was also experiencing homelessness, we included individuals with distinctly high service use, who likely differed from individuals in the Canadian study population. Furthermore, ED visits among unhoused individuals occasionally may be motivated by social needs, such as need for food or shelter,^50^ which may not necessitate an admission. Corroborating this possibility, a recent study of the top 10% of HUMS found that those who received a shelter-in-place hotel placement had significantly fewer ED visits.^51^

Our findings have several implications. First, understanding natural trends in health care use among high users can help focus interventions. Although our study examined health care use trends after initial high use, future studies might elucidate patient and structural characteristics preceding high use to determine how to prevent it altogether. Second, the decrease in ED visits among high users in San Francisco during the COVID-19 pandemic implies that there was some pandemic-specific factor(s) associated with decreased ED use. Potential candidates include shelter-in-place hotels and/or managed alcohol programs, which delivered integrated medical and behavioral health services to people experiencing homelessness.^38,39,51,52^ Although investigating the association of these services with ED use was beyond the scope of this study, future work might use similar methods to evaluate specific interventions. Another potential explanation for the observed decrease in use might have been fear of contracting COVID-19 in the ED. A qualitative study interviewing high users and their feelings toward health care during the COVID-19 pandemic could explore this possibility further.

### Limitations

This study has some limitations. Given the structure of CCMS data, we were only able to analyze ED visits on a FY basis, which did not align perfectly with the beginning of the COVID-19 pandemic and inhibited our ability to describe microtrends during the various waves of the pandemic. Furthermore, because CCMS was limited to San Francisco County, analysis of complete use patterns of patients who also received services in neighboring counties was precluded, potentially leading to the underestimation of total health care use. Although these limitations might be expected to restrict power, we still detected a statistically significant negative association between ED use and the pandemic—lending credence to our study’s conclusions.

Another limitation of our study was loss to follow-up. The mean loss to follow-up was approximately 19%, with earlier cohorts having higher loss to follow-up than later cohorts. We were unable to ascertain whether these individuals moved away from San Francisco or simply stopped using services. However, sensitivity analysis assuming all participants lost to follow-up stopped using services did not qualitatively change our results.

In addition, our study’s generalizability may be limited by San Francisco’s unique population and response to the COVID-19 pandemic. Our study population had high rates of homelessness, substance use, mental health diagnoses, and public insurance. During the pandemic, the city created novel services (eg, isolation/quarantine hotels, managed alcohol programs, and shelter-in-place hotels) to support the needs of this population. Nevertheless, high users of health care services exist nationwide, with documented high rates of homelessness,^4,8^ substance use disorder,^9,10^ chronic mental illness,^1,10^ and public insurance.^5,12,13,14,15^ Thus, our study’s findings likely apply to many subsets of high users and may support the need for innovative services aimed at addressing social needs.

## Conclusions

In this cohort study, among the top 5% of HUMS in San Francisco County, we observed a significant decrease in ED visits during the COVID-19 pandemic, even beyond a natural decay in use over time. The same techniques used in our study could be used to evaluate whether a specific intervention is associated with decreased health care use among high users. Further research is needed to elucidate COVID-19 pandemic–specific factors associated with the observed decrease in ED use and to understand how this change in use may have affected health outcomes. Identifying such factors could help inform interventions aimed at reducing ED visits and improving comprehensive care for this vulnerable population.

## Notes

Supplement. **eTable 1. **CCMS Urgent/Emergent Service Use Data Organization **eTable 2. **Observed Characteristics Between Participants with Complete versus Incomplete Data **eFigure 1. **Trend in Average Emergency Department Visits in Each Cohort Over Time, Aligned by Year of Follow Up **eFigure 2. **Average ED Visits Predicted by Negative Binomial Regression, by Time and COVID Year
