# Supplementary material for: Analysis of Emergency Department Encounters Among High Users of Health Care and Social Service Systems Before and During the COVID-19 Pandemic
Source: JAMA Netw Open. 2022 Oct 28;5(10):e2239076. doi: 10.1001/jamanetworkopen.2022.39076 (PMC9617170; doi:10.1001/jamanetworkopen.2022.39076)
Supplement: Supplement. — eTable 1. CCMS Urgent/Emergent Service Use Data Organization eTable 2. Observed Characteristics Between Participants with Complete versus Incomplete Data eFigure 1. Trend in Average Emergency Department Visits in Each Cohort Over Time, Aligned by Year of Follow Up eFigure 2. Average ED Visits Predicted by Negative Binomial Regression, by Time and COVID Year [file jamanetwopen-e2239076-s001.pdf]

Supplementary Online Content

Molina M, Evans J, Montoy JC, et al. Analysis of emergency department encounters among high users of health care and social service systems before and during the COVID-19 pandemic. *JAMA Netw Open*. 2022;5(10):e2239076. doi:10.1001/jamanetworkopen.2022.39076

eTable 1. CCMS Urgent/Emergent Service Use Data Organization

eTable 2. Observed Characteristics Between Participants with Complete versus Incomplete Data

eFigure 1. Trend in Average Emergency Department Visits in Each Cohort Over Time, Aligned by Year of Follow Up

eFigure 2. Average ED Visits Predicted by Negative Binomial Regression, by Time and COVID Year

This supplementary material has been provided by the authors to give readers additional information about their work.

© 2022 Molina M et al. *JAMA Network Open.*

eTable 1. CCMS Urgent/Emergent Service Use Data Organization

**Medical Urgent/Emergent Services**
Medical Hospital STAYS (via ZSFG and Out of Medical Group providers)
ED VISITS (via ZSFG and Out of Medical Group providers)
Medical Urgent Care VISITS (ZSFG Urgent Care, Tom Waddell Urgent Care)
**Psychiatric/Mental Health Urgent/Emergent Services**
MH Hospital STAYS (ZSFG)
PES VISITS (ZSFG)
MH Urgent Care VISITS (Dore Urgent Care)
**Substance Use Urgent/Emergent Services**
Sobering Center VISITS
SUD Medical Detox STAYS
SUD Social Detox STAYS

Abbreviations: CCMS=Coordinated Care Management System; ZSFG=Zuckerberg San Francisco General; ED=emergency department; MH=mental health; PES=psychiatric emergency services; SUD=substance use disorder

© 2022 Molina M et al. *JAMA Network Open.*

eTable 2. Observed Characteristics Between Participants with Complete versus Incomplete Data

	Complete	Incomplete			
		Lost to Follow Up	*P* Value[a]	Died	*P* Value[b]
**Characteristic**	**N (%)**	**N (%)**		**N (%)**	
**Age in yrs, mean (SD)**	45.7 (14.0)	45.0 (13.9)	0.13	55.1 (12.8)	<0.01
**Gender**					
Women	1,939 (31.7)	511 (29.6)	0.19	364 (32.4)	0.46
Men	4,117 (67.3)	1,200 (69.6)		754 (67.0)	
TG/Other/Declined	61 (1.0)	14 (0.8)		7 (0.6)	
**Language**					
English	5,399 (88.3)	1,562 (90.6)	<0.01	971 (86.3)	<0.01
Spanish	519 (8.5)	100 (5.8)		80 (7.1)	
Other	199 (3.3)	63 (3.7)		74 (6.6)	
**Race and Ethnicity**					
White	2,102 (34.4)	763 (44.2)	<0.01	424 (37.7)	<0.01
AA / Black	2,125 (34.7)	512 (29.7)		368 (32.7)	
Latinx	1,137 (18.6)	215 (12.5)		161 (14.3)	
Other	719 (11.8)	175 (10.1)		152 (13.5)	
Declined	34 (0.6)	60 (3.5)		20 (1.8)	
**Homelessness**[c]					
No	1,253 (20.5)	339 (19.7)	0.45	333 (29.6)	<0.01
Yes	4,864 (79.5)	1,386 (80.4)		792 (70.4)	
**Last Insurance Status**					
Medicaid - Non SSI	2,901 (47.4)	644 (37.3)	<0.01	334 (29.7)	<0.01
Medicaid With SSI/ Medi-Medi	2,687 (43.9)	684 (39.7)		582 (51.7)	
Medicare Only	281 (4.6)	96 (5.6)		90 (8.0)	
Other[d]	248 (4.1)	301 (17.5)		119 (10.6)	
**Jail Stay**[e]					
No	4,596 (75.1)	1,371 (79.5)	<0.01	967 (86.0)	<0.01
Yes	1,521 (24.9)	354 (20.5)		158 (14.0)	
**MH Diagnosis**					
No	2,261 (38.5)	635 (38.8)	0.83	554 (49.8)	<0.01
Yes	3,613 (61.5)	1,002 (61.2)		558 (50.2)	
**SUD Diagnosis**					
No	1,834 (31.2)	586 (35.8)	<0.01	371 (33.4)	0.16
Yes	4,040 (68.8)	1,051 (64.2)		741 (66.6)	

Abbreviations: yrs=Years; FY1213=Fiscal Year 2012-2013; SD=standard deviation; TG=transgender; AA=African-American; SSI=Supplemental Security Income; Medi-Medi=Medicaid/Medicare; MH=mental health; SUD=substance use disorder

[a]Comparing characteristics in those lost to follow up to those with complete follow up; χ^2 used to evaluate categorical variables, Kruskal-Wallis used to evaluate continuous variables

[b]Comparing characteristics in those who died to those with complete follow up; same tests used as above

[c]History of homelessness

[d]Other included uninsured, private insurance, Healthy San Francisco, or other insurers

[e]Within past fiscal year

© 2022 Molina M et al. *JAMA Network Open.*

eFigure 1. Trend in Average Emergency Department Visits in Each Cohort Over Time, Aligned by Year of Follow Up

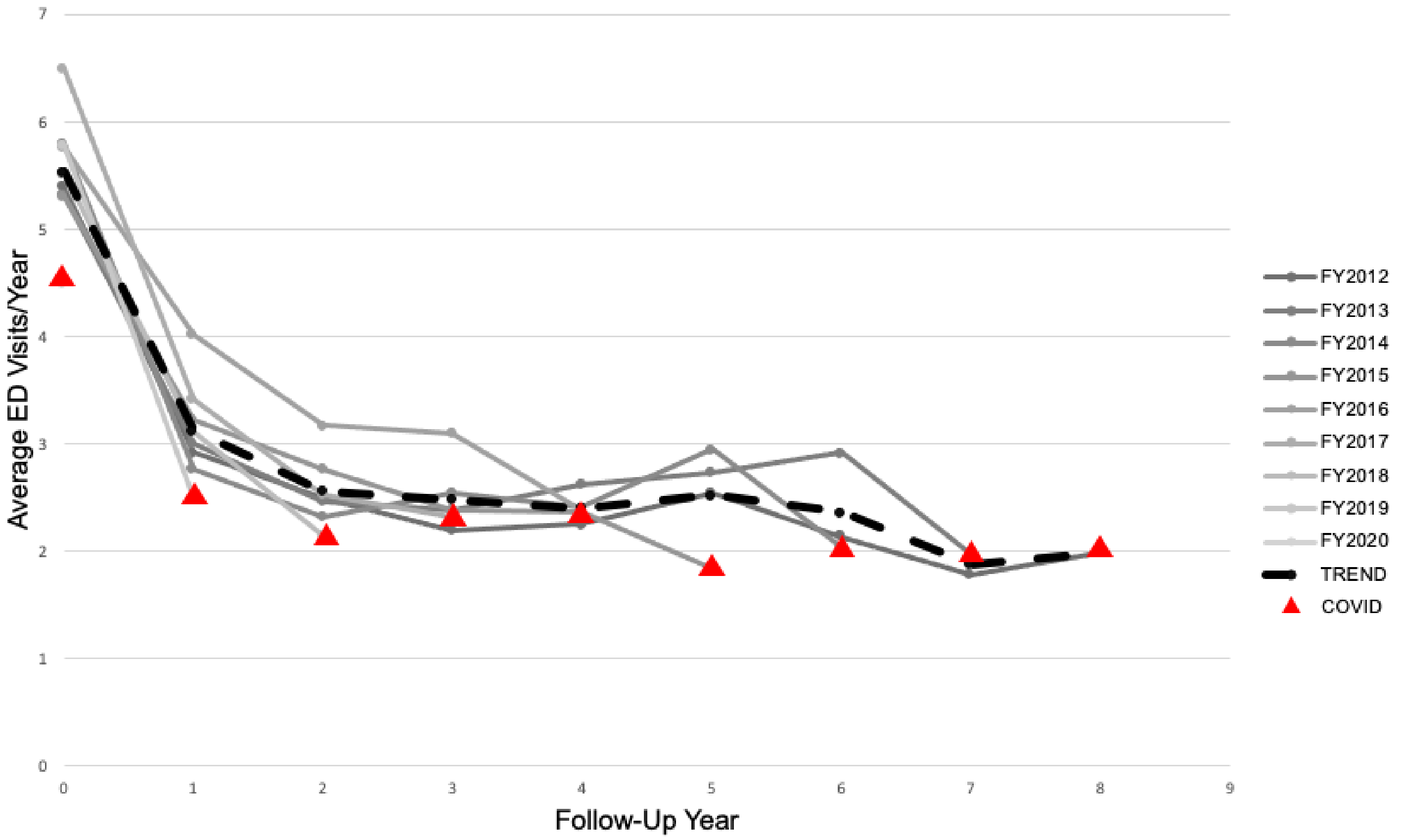

Abbreviations: ED=Emergency Department, FY=Fiscal Year, COVID=COVID-19 Year
Caption: Cohorts have been “shifted left” and aligned by year of follow up. In each cohort, average annual ED visits decreased over time, highlighted by the overall (black, dashed) trend line. However, during each respective cohort’s COVID-19 year (red triangle), ED visits were on average lower than that expected by the overall trend.

© 2022 Molina M et al. *JAMA Network Open.*

eFigure 2. Average ED Visits Predicted by Negative Binomial Regression, by Time and COVID Year

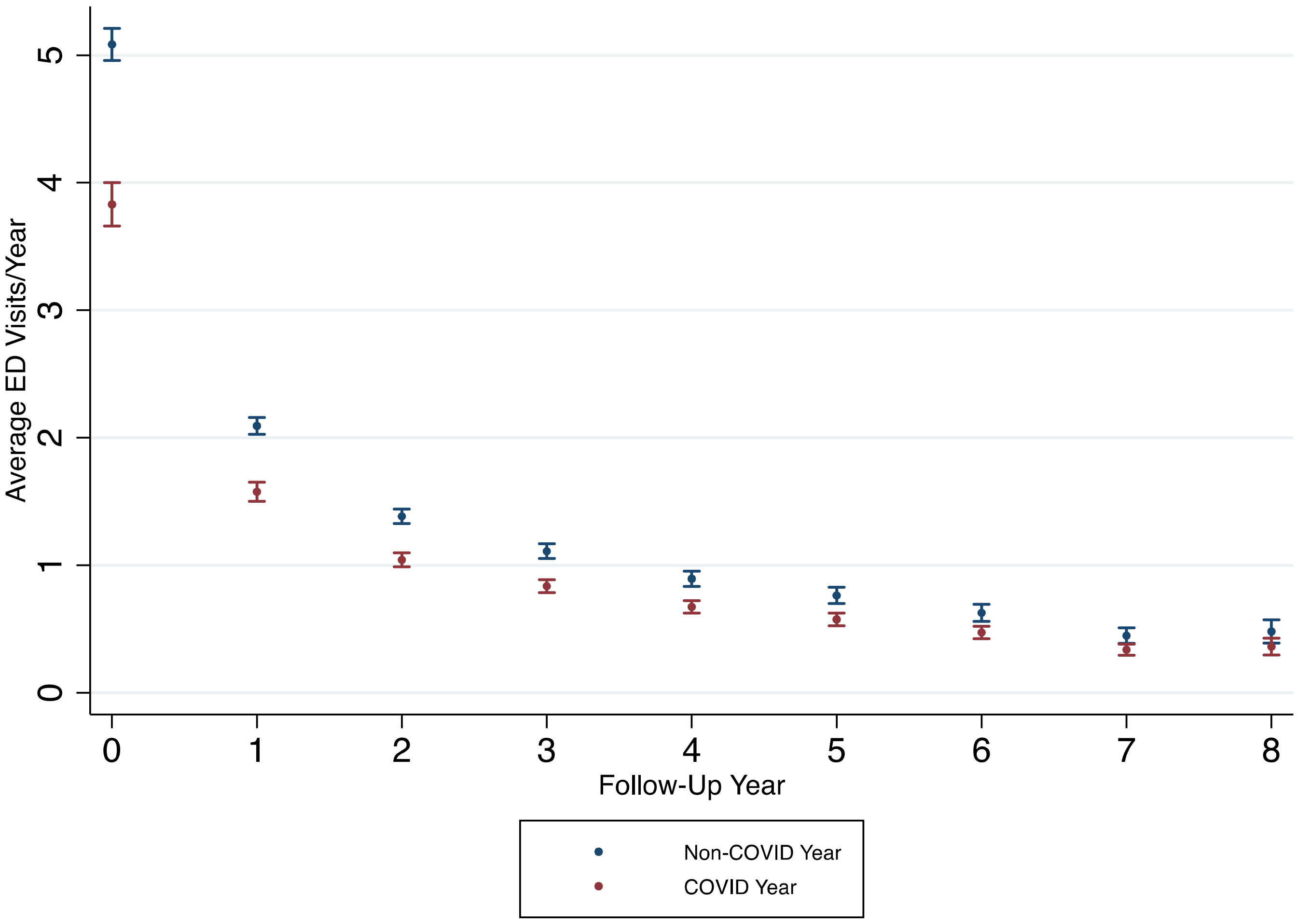

© 2022 Molina M et al. *JAMA Network Open.*
